# Nucleoside Reverse Transcriptase Inhibitors Are the Major Class of HIV Antiretroviral Therapeutics That Induce Neuropathic Pain in Mice

**DOI:** 10.3390/ijms25169059

**Published:** 2024-08-21

**Authors:** Keegan Bush, Yogesh Wairkar, Shao-Jun Tang

**Affiliations:** 1 Department of Neuroscience and Cell Biology, University of Texas Medical Branch, Galveston, TX 77555, USA; kmbush@utmb.edu; 2 Department of Neurology, University of Texas Medical Branch, Galveston, TX 77555, USA; 3 Stony Brook University Pain and Analgesia Research Center and Department of Anesthesiology, Renaissance School of Medicine, Stony Brook University, Stony Brook, NY 11794, USA

**Keywords:** HIV, antiretroviral drugs, nucleoside reverse transcriptase inhibitors, pain, neuropathy

## Abstract

The development of combination antiretroviral therapy (cART) has transformed human immunodeficiency virus (HIV) infection from a lethal diagnosis into a chronic disease, and people living with HIV on cART can experience an almost normal life expectancy. However, these individuals often develop various complications that lead to a decreased quality of life, some of the most significant of which are neuropathic pain and the development of painful peripheral sensory neuropathy (PSN). Critically, although cART is thought to induce pain pathogenesis, the relative contribution of different classes of antiretrovirals has not been systematically investigated. In this study, we measured the development of pathological pain and peripheral neuropathy in mice orally treated with distinct antiretrovirals at their translational dosages. Our results show that only nucleoside reverse transcriptase inhibitors (NRTIs), not other types of antiretrovirals such as proteinase inhibitors, non-nucleoside reverse transcriptase inhibitors, integrase strand transfer inhibitors, and CCR5 antagonists, induce pathological pain and PSN. Thus, these findings suggest that NRTIs are the major class of antiretrovirals in cART that promote the development of neuropathic pain. As NRTIs form the essential backbone of multiple different current cART regimens, it is of paramount clinical importance to better understand the underlying mechanism to facilitate the design of less toxic forms of these drugs and/or potential mitigation strategies.

## 1. Introduction

Antiretrovirals targeting human immunodeficiency virus (HIV) have revolutionized treatment for this deadly disease, and these drugs continue to be an active avenue of investigation, both to identify new compounds that inhibit viral replication and to better understand the secondary symptoms they induce in patients. Combination antiretroviral therapy (cART) effectively inhibits HIV replication in patients, drastically increasing life expectancy post-infection [1]. However, although the profile of HIV-associated neurocognitive disorder (HAND) has shifted away from dementia and severe neurocognitive disorder in the cART era [2], the prevalence of peripheral sensory neuropathy (PSN) has remained unchanged at around 30–60%, with the variation likely arising from different assessment methods and variable inclusion of decreased sensation neuropathy [3,4,5]. The leading hypothesis to explain these trends proposes that cART is involved in the initiation and perpetuation of neuropathic symptoms [2,6,7,8,9,10,11].

Many studies have demonstrated the potential for several cART components to inhibit neuronal function and induce neurodegeneration. However, these neurotoxic effects are highly dependent on antiretroviral classification [12,13,14,15,16,17,18,19,20,21,22,23,24,25,26,27,28,29,30,31,32,33,34,35,36]. In particular, nucleoside reverse transcriptase inhibitors (NRTIs) are the primary component of cART implicated in PSN development [12,36,37,38,39]. These were the first class of antiretrovirals to be used for HIV [10] and remain one of the most efficacious drugs for long-term treatment of HIV-positive patients. Thus, despite their known detrimental effects, the use of NRTIs in patients cannot be discontinued.

Other classes of cART therapeutics include non-nucleoside reverse transcriptase inhibitors (NNRTIs); viral entry inhibitors, which can be separated into C-C chemokine receptor type 5 antagonists (CCR5As) and fusion inhibitors (FIs); integrase strand transfer inhibitors (INSTIs), sometimes referred to as integrase inhibitors (IIs); and protease inhibitors (PIs). In general, NNRTIs are one of the most well-tolerated components of cART, with few side effects or drug–drug interactions [9,10,40]. One exception, however, is efavirenz, which has been shown to induce sleep disorders and detrimental psychiatric symptoms in patients and to promote neurodegenerative effects in vitro and in vivo [20,31,32,33,34]. In addition, NNRTIs display one of the lower barriers to resistance among cART components [41,42]. Viral entry inhibitors also tend to be well tolerated, although they carry a slight risk of hepatotoxicity [13,43,44]. However, the injectable nature of FIs has limited their use. This is especially true of enfuvirtide, which requires bis in die (twice per day) injection. In contrast, ibalizumab, a newer FI, only requires injection once per week or even once every two weeks and therefore may be more accessible to patients [14,15,16,45,46,47]. INSTIs are the second most utilized cART component, often prescribed with two NRTIs for an initial cART regimen [17,48,49,50,51]. INSTIs also tend to be relatively well tolerated, with some evidence linking them to insomnia in patients [18,19]. On the other hand, PIs display a variable array of off-target effects, with evidence linking these drugs to the development of insulin resistance, increased risk of heart disease, lipodystrophy, and hepatotoxicity, as well as a high potential for drug–drug interaction [23,28,52,53,54,55,56]. Notably, although there is no direct evidence for PIs inducing PSN at clinically relevant levels, some have speculated they may promote the exacerbation of NRTI-induced neuropathy [26,27].

In numerous studies, these various drug classes have been investigated in vivo and in vitro to determine and confirm their effects, many of which have recapitulated observed outcomes in patients. However, a number of animal models used to test the nociceptive effects of various cART components have utilized injection as the medium for administration. For FIs, this dosing method mimics patient administration; however, NRTIs, NNRTIs, CCR5As, and PIs are administered as ingested pills. Thus, the use of injection administration in these animal models excludes the effects of first-pass metabolism, and critically, several lines of evidence suggest that the secondary metabolites of these drugs are involved in their secondary effects [10,23,33,52,57].

Although many preclinical studies have been carried out to determine the neurotoxicity of various cART components, these previous studies were often performed under diverse experimental conditions, such as using different animal models or administering drugs from different routes. Consequently, results from such studies are not ideal for comparisons to evaluate the relative contribution of cART components to neuropathy or other types of neurotoxicity. To address this problem, we carried out, to our knowledge, the first study to systematically compare the neurotoxicity of different classes of cART components in inducing neuropathy in the same mouse model. To mimic the oral administration of drugs by HIV patients on cART, we developed a water-based ingestion regime for mice at the translated equivalent dose of each drug class, which was based on methods from past studies utilizing corresponding water treatment methods [58,59,60]. We measured the thermal and mechanical nociception profile of each mouse throughout the 4-week dosing regimen, followed by sacrifice and tissue acquisition for further processing. We found that treatment with NRTIs induces an increase in nociceptive sensitivity as well as a decrease in epidermal innervation of the hind paw. These neuropathic phenotypes mimic patient PSN presentation, suggesting that drug-induced neuropathy translates well in our mouse model [3,4,61]. In contrast, the representative NNRTI, CCR5A, and PI drugs did not induce nociceptive or epidermal innervation changes compared to the control. Notably, the close recapitulation of observed patient responses to these compounds observed in this study suggests that our ingested administration model may be utilized to further investigate the secondary effects of cART components in future studies.

## 2. Results

### 2.1. cART Induces Mechanical Nociception Changes

Previous studies using mammalian animal models have reported mechanical nociception changes in response to NRTI and PI administration [27,36,53,62,63,64]. However, in many of these investigations, the drugs were administered via injection; furthermore, several of the NRTI studies and all of the PI studies that detected nociception changes used dosing regimens that translate above clinically relevant levels. Thus, although these studies have reported neurodegenerative effects, they do not mimic patient administration conditions. Here, utilizing a mouse model with clinically relevant translated doses and oral drug administration, we hypothesized that mice treated with an NRTI would exhibit a decreased mechanical nociceptive threshold, whereas in those administered a PI, II, CCR5 antagonist, or NNRTI, the exhibited mechanical nociception would remain constant.

**Figure 1:**
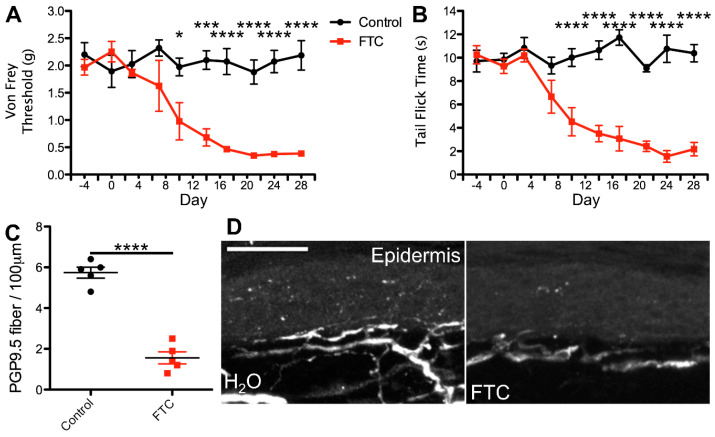
Nucleoside reverse transcriptase inhibitor (NRTI) orally administered at the translated patient dosage induces nociception sensitization and epidermal denervation in mice. (**A**) Average von Frey filament threshold in grams throughout the 4-week treatment regimen for mice administered 0.098 mg/mL emtricitabine (FTC; concentration used in all experiments), a commonly used NRTI, in water and control animals (*n* = 5, both groups). (**B**) Average tail flick times in seconds throughout the 4-week treatment regimen for FTC-treated and control mice. (**C**) Number of PGP9.5-labeled axons per 100 μm, averaged from 10 sections per mouse, in FTC-treated and control mice. (**D**) Representative images of PGP9.5-labeled axons in hind paw glabrous skin epidermis from FTC-treated and control mice. * *p* < 0.05, *** *p* < 0.001, **** *p* < 0.0001; error bars show the standard error of the mean (S.E.M.); scale bar, 50 µm.

**Figure 2:**
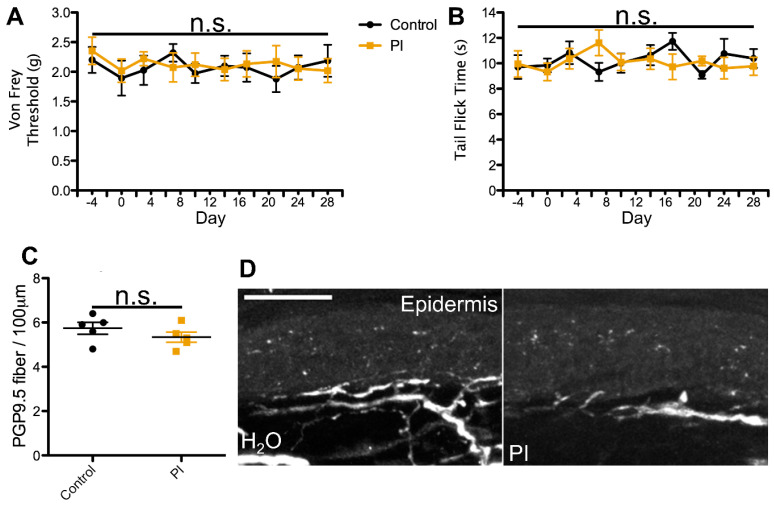
Protease inhibitor (PI) orally administered at the translated patient dosage does not induce changes in nociception or epidermal innervation in mice. (**A**) Average von Frey filament threshold in grams throughout the 4-week treatment regimen for mice administered 0.59 mg/mL ritonavir (PI; concentration used in all experiments) in water and control animals (*n* = 5, both groups). (**B**) Average tail flick times in seconds throughout the 4-week treatment regimen for ritonavir-treated and control mice. (**C**) Number of PGP9.5-labeled axons per 100 μm, averaged from 10 sections per mouse, in ritonavir-treated and control mice. (**D**) Representative images of PGP9.5-labeled axons in hind paw glabrous skin epidermis from ritonavir-treated and control mice. No significant differences (n.s.) in nociception or epidermal innervation relative to the control were observed at any time point. Error bars show the S.E.M.; scale bar, 50 µm.

**Figure 3:**
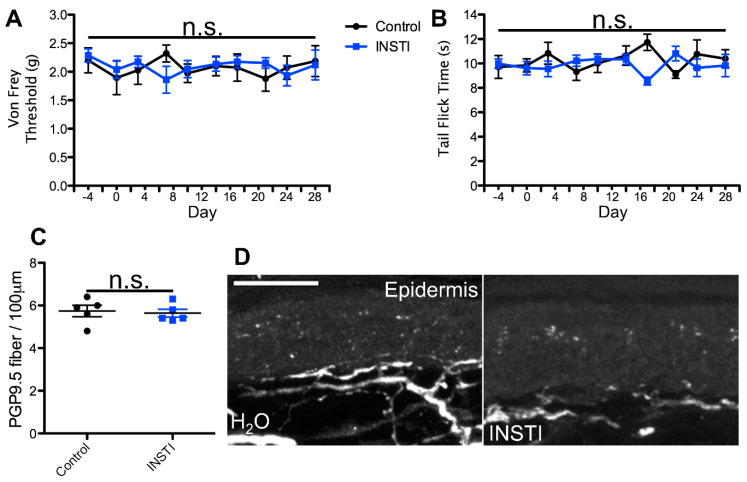
Integrase strand transfer inhibitor (INSTI) orally administered at the translated patient dosage does not induce changes in nociception or epidermal innervation in mice. (**A**) Average von Frey filament threshold in grams throughout the 4-week treatment regimen for mice administered 0.39 mg/mL raltegravir (INSTI; concentration used in all experiments) in water and control animals (*n* = 5, both groups). (**B**) Average tail flick times in seconds throughout the 4-week treatment regimen for raltegravir-treated and control mice. (**C**) Number of PGP9.5-labeled axons per 100 μm, averaged from 10 sections per mouse, in raltegravir-treated and control mice. (**D**) Representative images of PGP9.5-labeled axons in hind paw glabrous skin epidermis from raltegravir-treated and control mice. No significant differences (n.s.) in nociception or epidermal innervation relative to the control were observed at any time point. Error bars show the S.E.M.; scale bar, 50 µm.

**Figure 4:**
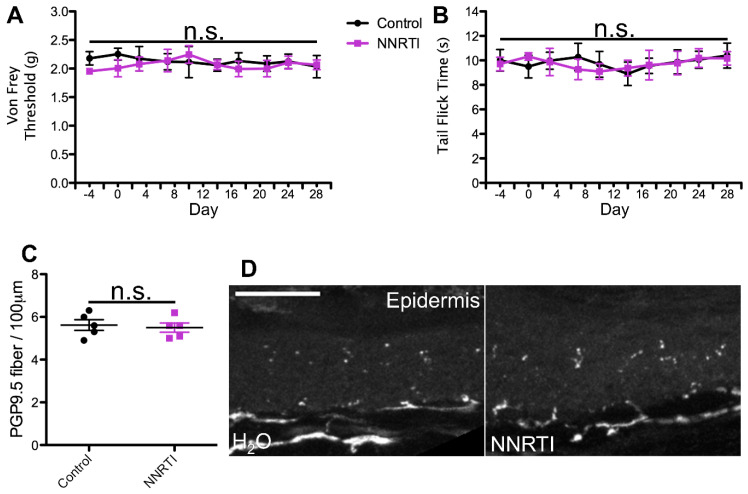
Non-nucleoside reverse transcriptase inhibitor (NNRTI) orally administered at the translated patient dosage does not induce changes in nociception or epidermal innervation in mice. (**A**) Average von Frey filament threshold in grams throughout the 4-week treatment regimen for mice administered 0.29 mg/mL efavirenz (NNRTI; concentration used in all experiments) in water and control animals (*n* = 5, both groups). (**B**) Average tail flick times in seconds throughout the 4-week treatment regimen for efavirenz-treated and control mice. (**C**) Number of PGP9.5-labeled axons per 100 μm, averaged from 10 sections per mouse, in efavirenz-treated and control mice. (**D**) Representative images of PGP9.5-labeled axons in hind paw glabrous skin epidermis from efavirenz-treated and control mice. No significant differences (n.s.) in nociception or epidermal innervation relative to the control were observed at any time point. Error bars show the S.E.M.; scale bar, 50 µm.

**Figure 5:**
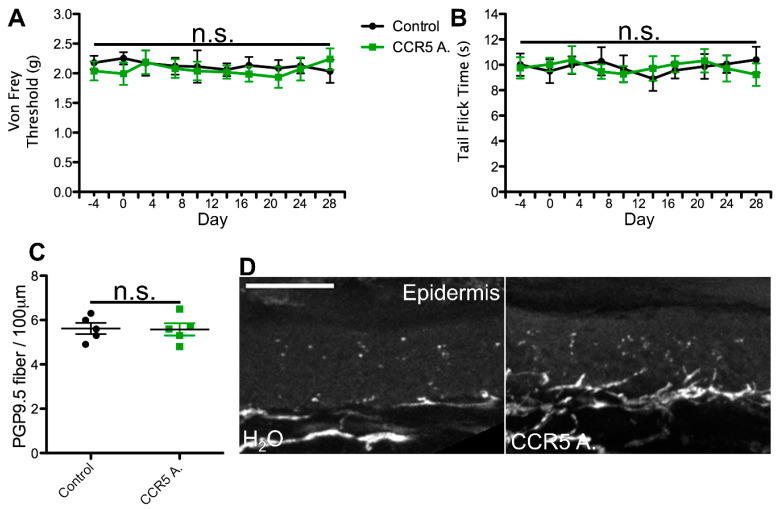
C-C chemokine receptor type 5 antagonist (CCR5A) orally administered at the translated patient dosage does not induce changes in nociception or epidermal innervation in mice. (**A**) Average von Frey filament threshold in grams throughout the 4-week treatment regimen for mice administered 0.29 mg/mL maraviroc (CCR5A; concentration used in all experiments) in water and control animals (*n* = 5, both groups). (**B**) Average tail flick times in seconds throughout the 4-week treatment regimen for maraviroc-treated and control mice. (**C**) Number of PGP9.5-labeled axons per 100 μm, averaged from 10 sections per mouse, in maraviroc-treated and control mice. (**D**) Representative images of PGP9.5-labeled axons in hind paw glabrous skin epidermis from maraviroc-treated and control mice. No significant differences (n.s.) in nociception or epidermal innervation relative to the control were observed at any time point. Error bars show the S.E.M.; scale bar, 50 µm.

To test this hypothesis, we utilized a previously developed model for azidothymidine (AZT) administration via the water route as a framework for our dosing regimens. This study reported that administration of AZT causes no visible distress to the animals and no changes in water intake, and the drug shows stability in water [58,59,60]. Using the known translation index for mice by weight, combined with the average water intake of mice, we then translated the most commonly administered patient dose for each cART component. For the NRTI and CCR5 antagonist, we administered the most commonly prescribed variant, whereas for the II, PI, and NNRTI, we administered the therapeutic variant that exhibits the most neurotoxic potential. This decision was made based on the notably enhanced neurodegenerative potential demonstrated by one form of these drugs relative to others in the same class [18,20,31,32,33,34,52,53,57] and our goal of testing the PSN-inducing ability of the worst-case patient therapeutic regimen for each cART component. In this model, mice ingested individual cART components over a period of 4 weeks and were monitored for mechanical nociceptive changes throughout their treatment via von Frey filament testing. Consistent with our predictions, we detected a progressive decrease in mechanical nociceptive threshold over a period of 3 weeks and a near steady allodynia-like phenotype for the last week of treatment in mice administered the NRTI (FTC)(Figure 1A). In contrast, mice treated with a PI, II, NNRTI, or CCR5 antagonist exhibited no deviation in mechanical nociception throughout the 4-week treatment regimen, relative to controls (Figure 2, Figure 3, Figure 4 and Figure 5A). This suggests that solely NRTIs show greater potential for the induction of mechanical sensitization when given under conditions that closely mimic those for patient administration.

### 2.2. Thermal Nociception Sensitization Induced by cART

HIV patients with PSN report an array of pain conditions, including numbness, increased sensitivity to touch or thermal extremes, and even, in some cases, chronic pain [3,4,65]. These varied pain profiles likely result from a number of factors; however, reports of PSN linked to NRTIs tend to involve mechanical and thermal components. Therefore, we hypothesized that changes in thermal nociception occurring in response to ingestion of individual cART components in our model would mirror any mechanical nociception changes.

To test this possibility, we monitored all mice for any alterations in thermal nociception throughout the 4-week treatment regimen using the hot water bath tail flick test [66,67]. This method of thermal nociception assessment was chosen over the more commonly used hot plate test due to our need for continued use, as repeated hot plate testing may induce tissue damage and skew the results. In addition, this method of thermal nociception assessment utilizes a different appendage than the hind paw, which is used in von Frey testing, potentially providing a more generalized insight into the mouse nociception profile in response to cART. As hypothesized, we observed no changes in thermal nociception over the 4-week dosing regimen in mice administered a PI, II, CCR5A, or NNRTI relative to control animals (Figure 2, Figure 3, Figure 4 and Figure 5B). Conversely, mice administered the NRTI (FTC) displayed a faster response to the fixed temperature water bath, suggesting an increased thermal sensitization that occurs in parallel with the changes in mechanical nociception described above (Figure 1B). Thus, these data indicate that the cART component implicated in the development of mechanical nociception sensitization also induces changes in thermal nociception in an ingestion mouse model.

### 2.3. Epidermal Denervation Induced by cART

HIV patients with PSN have been examined for changes in epidermal innervation, with neuropathy and epidermal neuron loss observed in some cases [61,68,69,70]. Therefore, we hypothesized that cART components that induce changes in nociception profiles would also promote denervation of the epidermis. To test this possibility, we harvested glabrous hind paw skin tissue at the conclusion of our 4-week cART administration regimen and measured epidermal innervation via PGP9.5 staining. Our data revealed relatively consistent innervation in mice treated with the control, II, PI, CCR5A, and NNRTI (Figure 2, Figure 3, Figure 4 and Figure 5C,D), whereas we detected a significant decrease in epidermal axon counts in NRTI-treated mice (Figure 1C,D). This suggests that patterns of epidermal peripheral neuron degeneration occurring in response to oral NRTI administration in mice may model those observed in PSN patients. Overall, our findings suggest that this ingestion model may show utility for closely modeling and uncovering the mechanism for NRTI-induced PSN-associated pain in patients.

## 3. Discussion

In this study, we measured the effects of individual cART therapeutics, ingested at the translated human dosage equivalent, on nociception and peripheral neurodegeneration in a mouse model. Critically, this water ingestion model more closely mimics the method for patient administration than previous studies that used injection-based administration [27,36,53,58,59,60,62,63,64]. We found that when orally administered at clinically relevant levels, the PI, II, CCR5A, and NNRTI did not induce detectable changes in nociception and epidermal innervation. In contrast, ingestion of the NRTI led to a maintained thermal and mechanical allodynia-like phenotype, with associated loss of epidermal axon staining. We further note that, to the best of our knowledge, this study is the first to assess nociception and peripheral neurodegeneration in response to ingestion of cART therapeutics in a mouse model.

### 3.1. Ingestion of cART

In HIV patients, one of the most common cART regimens combines two NRTIs and an INSTI [1,17,48,49,50,51], a formula that is utilized for its efficacy in reducing viral load and the low potential for both drug–drug interactions and the development of HIV resistance. However, it is not applicable in all cases, as some individuals exhibit severe acute adverse reactions to this formulation. In such instances, alternative combinations are tested until a well-tolerated and effective regimen is found. Regardless, even for these rare cases, there is a tendency towards the use of therapeutics that are easily administered by patients to promote compliance [14,15,16,45,46,47], which precludes the use of injected cART, if avoidable. Therefore, an animal model that mimics the most common method for patient administration would represent a powerful tool for investigating the effects of cART and the physiological response to these therapeutics. As such, a key aim of our study was to investigate the potential viability and translation relevance of a cART oral administration mouse model.

The inherent difference between ingestion and injection administration primarily derives from two features. The first is the potential effects from first-pass metabolism with ingested drugs, which is avoided in injection. Although the metabolic profiles of many individual cART component variants have not been well characterized, it was shown that PIs are readably metabolized by the CYP3A pathway [52], which contributes to their drug–drug interactions. In addition, there is evidence that at least one NNRTI metabolite exhibits potential for increased neurotoxicity in vitro [9,10,23,33,52,57,71]. Based on these observations, a model that includes the first-pass metabolism of cART components is likely to better recapitulate patient conditions. The second major source of variation between ingestion and injection relates to the relative dynamic pharmacokinetics induced by these distinct forms of drug administration [72,73,74,75,76,77,78]. That is, while ingestion may induce a bolus of drug above the target dosage absorption, this usually occurs to a much lesser extent relative to injection administration. Thus, injection may show increased potential for effects, such as above-target dosages and both off-target and toxic effects compared to ingestion. We therefore expect that our ingestion model has less potential to generate false positives resulting from such effects, again suggesting closer modeling of the patient condition than injection.

### 3.2. Neurotoxicity of cART

The possible neurotoxicity of many of cART components has been tested in vitro and in several animal models. In particular, some PI variants have been shown to induce behavioral deficits, and there is further evidence for PI-induced neurodegeneration in neuron cultures. However, many of these studies used older PI dosing regimens that were known to induce CNS effects in patients, whereas improved protocols for cotreatment with pharmacokinetic enhancers have lowered the effective dosage for PI treatment [27,28,53,79]. Thus, PI concentrations shown to promote neurotoxicity are supraphysiological relative to current patient regimens [10]. Consistent with this, we detected no nociceptive or epidermal denervation effects from the ingested PI at the current translated dosage in our mouse model. In this context, it is important to note that an early study reported indinavir to induce peripheral neuropathy in a rat model [80]. It will be interesting to examine in future studies if this PI causes neuropathy in the mouse model used in the current study.

IIs are generally considered well tolerated and non-neurotoxic, with two exceptions. Raltegravir has been linked to insomnia and anxiety in patients, with in vitro studies demonstrating potential for the induction of increased stress responses at supraphysiological concentrations [18,20]. Secondly, and conversely, studies on dolutegravir are mixed, with some evidence linking it to the development of similar neuropsychiatric symptoms, while other evidence suggests that this compound exhibits a potential to inhibit neurodegeneration in the CNS [81,82].

Similar to IIs, most NNRTIs exhibit few to no neurodegenerative effects in patients. The one exception to this is efavirenz, which has been linked to insomnia and the development of severe neuropsychiatric disorders. In addition, several animal and in vitro studies have demonstrated significant efavirenz-induced neurotoxicity, with evidence for mitochondrial dysfunction [20,31,34], although we note that these studies did not assess the potential for peripheral neurodegeneration or measure nociception profiles in response to efavirenz.

Similarly, there have been no studies reporting neurotoxic effects from CCR5A administration. In contrast, there is some evidence that intrathecal-injected CCR5A exhibited analgesic effects in a sciatic nerve constriction model, with associated downregulation of pro-inflammatory markers in microglia [83].

Distinct from other cART therapeutics, NRTIs are notorious for inducing PSN in patients and in animal models. In particular, there is evidence that several forms of these drugs, including the more recently developed “non-neurotoxic” NRTIs in therapeutic use today, induce painful sensory neuropathy in male and female patients [9,12,35,36,84,85]. However, while NRTI-induced nociception and oral administration have been investigated in animal models individually, to the best of our knowledge, there are no previous reports of nociception changes in response to ingested NRTIs in animals. Therefore, our study is the first to show evidence for nociceptive sensitization and peripheral neurodegeneration induced in response to oral treatment with NRTIs in a mouse model.

## 4. Materials and Methods

### 4.1. Animals

These experiments were performed with adult C57BL/6J mice (10–18 weeks old and weighing 20–32 g) purchased from The Jackson Laboratory (Bar Harbor, ME, USA). All experimental procedures were approved by the Institutional Animal Care and Use Committee at the University of Texas Medical Branch (Protocol #1804026). Thermal and mechanical nociception testing was performed following the guidelines of the International Association for the Study of Pain. Mice were housed in cages (≤5 animals/cage) with standard bedding and free access to food and water, in a room maintained at 23 ± 3 °C under a 12/12 light–dark cycle.

### 4.2. Mouse Thermal Nociception

Mouse thermal nociception was assessed by tail flick testing, as described previously [66,67]. For these experiments, a water bath was heated to 48 °C with precise monitoring of the temperature, as even temperature fluctuations of 1–2 °C may significantly affect mouse response time. Mice were placed in plastic restraining cones with small open ends (smaller than the standard nose opening size) and allowed to adjust themselves until their tail protruded from the small cone end. Mice will normally self-insert their tails through the small opening; however, if they had trouble doing so, we assisted by either angling the cone slightly or retrying after slight enlargement of the cone tip opening. The larger cone section was then gently folded over the body and head of the mouse to restrain it. Note that the restraining cones were modified to have nose holes at the area of the head of the mouse for nose protrusion during the restrained steps. The mice were then positioned above the hot water bath, and a timer was started at the point of tail immersion. The timer was stopped upon tail twitch/flick or retraction, and the time was recorded. Note that in some instances, mice reacted prior to full tail immersion. In this case, we removed the tail from the water bath, let it rest in the air for 30 s, and retried. If tail flick was again observed prior to full tail submersion, the time was recorded as 0 s.

### 4.3. Mouse Mechanical Nociception

Mouse mechanical nociception was assessed by von Frey testing, as described previously [36,66]. Mice were habituated in 13.2 × 5 × 4 cm Plexiglas boxes for 1 h on three continuous days prior to behavioral testing. On testing days, the mice were first put in boxes for 20 min to minimize their exploring behavior. The test was then performed by applying the tip of a 3.61/0.4 g filament (Stoelting, Wood Dale, IL, USA) perpendicularly to the center plantar area of the mouse hind paw until the filament was slightly bent (roughly 30° from tip to tip) and remained bent for one second. Mouse response and filament switching were based on the up–down method. That is, if the mouse responded to the initial filament, we utilized the next smaller force filament, and if there was no response, we utilized the next larger force filament. This process was repeated until six up–down measurements were acquired per paw per mouse at each measurement time. Nociception withdraw response was recorded for abrupt paw withdrawal, lateral moving, shaking, lifting, licking, and/or biting of the touched area of the hind paw.

### 4.4. Mouse Treatment

Mouse treatment with cART was administered via water ingestion [58,59,60]. Each drug was thoroughly dissolved in an initial water volume of 100 mL, which was provided to the mice and refilled every day, or every other day (depending on the observed fill line), with freshly made 50 mL aliquots of the same drug, at the same relative concentration. The water bottles in the cages were closely monitored daily for ingestion and any precipitation of drug; all drugs were readily soluble at the administered doses, and no precipitation was observed. The concentrations for each cART component in water were as follows: 0.098 mg/mL emtricitabine (FTC; NRTI), 0.59 mg/mL ritonavir (PI), 0.39 mg/mL raltegravir (II/INSTI), 0.29 mg/mL efavirenz (NNRTI), and 0.29 mg/mL maraviroc (CCR5A); control mice received water without drug. At the average water consumption of 5 mL/day for mice of this age and weight, these concentrations roughly equate to the human equivalent of these drugs at their most common dosage regimens in patients. Water level changes were also monitored to confirm ingestion amounts. We detected no changes in water consumption within groups pre- and post-initiation of treatment or between the different treatment groups.

### 4.5. Mouse Dissection, Imaging, and Analysis

Dissection of the hind paw skin was performed as described previously [86,87]. Briefly, mice were anesthetized with 14% urethane (0.2–0.3 mL; intraperitoneally) and monitored for complete anesthetization via hind paw pinch prior to decapitation. After other dissection tissues were stored, hind paw skin was collected from both paws and placed in ice-cold 80% ethanol in phosphate-buffered saline (PBS) overnight (or until further processing). Skin sections were flat mounted on ice-cold gel plates and rehydrated in a series of ethanol solutions (50% EtOH, 30% EtOH, and 0% EtOH in PBS) prior to cryosectioning (40 μm) and staining with rabbit anti-PGP9.5. Neuronal innervation was quantified by manual counting of epidermal fibers in 100 μm long segments from 10 random sections located in a similar plantar area of each mouse; sections from both hind paws of individual mice were grouped together.

### 4.6. Statistical Analysis

Statistical analyses were performed using GraphPad Prism software (version 10.3.0). The significance of skin innervation data comprising single data points per mouse was analyzed using Student’s *t*-test for comparison between two groups and one-way analysis of variance (ANOVA) followed by the Bonferroni post hoc test for comparisons between multiple groups. The significance of the results from analyses yielding multiple data points per mouse (von Fray and tail flick data) was determined by two-way ANOVA, followed by the Bonferroni multiple comparisons test. Significance levels were determined using 95% confidence intervals, and *p*-values are indicated in the respective figure legends.

## 5. Conclusions

In this study, we utilized a newly developed mouse ingestion model with individual cART components to assess their nociceptive effects and measure epidermal neurodegeneration in response to these compounds. However, this model has several limitations. The first is that we administered only one component at a time, whereas in patients, cART commonly consists of three or more therapeutics used in conjunction [1,17,48,49,50,51]. This difference may lead to discrepancies between the spectrum of conditions exhibited by patients and our model. To address this concern, in future studies, we propose expanding our model to include the assessment of ingested cART combination regimens in order to investigate the effects of various drug combinations. Secondly, cART is administered in the presence of HIV infection, and the expression of viral products is a potential confounding factor in PSN development [6,88,89,90,91]. This can partially be addressed in future studies by testing the effects of oral cART administration in mouse models of HIV gp120 administration or in mice genetically engineered to express viral products [89,91,92]. A third limitation of our study is that an increased prevalence of PSN is observed in older patients [93,94,95]. This potential risk factor in cART administration can be addressed by performing studies with age-matched mice, which may more closely model this complication. Finally, patient conditions are not static, and often, with modification of cART regimens, PSN symptoms can resolve. Therefore, future investigations focusing on regimen modification of ingested cART may provide additional insights into the interplay between various therapeutics in the development and maintenance of PSN presentation. Overall, despite these limitations, this study has yielded a new, broadly applicable ingestion model for cART therapeutics that represents patient administration methods. Critically, this provides a valuable resource for future investigations into the physiological effects of cART treatment and provides a model with which to test possible therapeutic inhibition of cART-induced PSN in response to NRTI or cART administration.

Our results indicate that NRTIs are the major source of neurotoxicity in cART. These findings suggest that clinical practice dealing with the problems of neurotoxicity should consider effective cART regimens with no or different NRTIs. They also revealed that NRTIs are the top candidate for targeting the neurotoxicity of cART in translational research. With that said, we are cautious about the applicability of the findings to human conditions due to the potential limitations of mouse models in simulating the treatment of human HIV infection.

## Data Availability

The original contributions presented in this study are included in the article. Further inquiries can be directed to the corresponding author.

## References

[1] Saag M.S., Benson C.A., Gandhi R.T., Hoy J.F., Landovitz R.J., Mugavero M.J., Sax P.E., Smith D.M., Thompson M.A., Buchbinder S.P. (2018). Antiretroviral Drugs for Treatment and Prevention of HIV Infection in Adults. JAMA.

[2] Heaton R.K., Franklin D.R., Ellis R.J., McCutchan J.A., Letendre S.L., Leblanc S., Corkran S.H., Duarte N.A., Clifford D.B., Woods S.P. (2011). HIV-associated neurocognitive disorders before and during the era of combination antiretroviral therapy: Differences in rates, nature, and predictors. J. Neurovirol..

[3] Wulff E.A., Wang A.K., Simpson D.M. (2000). HIV-associated peripheral neuropathy: Epidemiology, pathophysiology and treatment. Drugs.

[4] Ghosh S., Chandran A., Jansen J.P. (2012). Epidemiology of HIV-related neuropathy: A systematic literature review. AIDS Res. Hum. Retroviruses.

[5] Sacktor N. (2002). The epidemiology of human immunodeficiency virus-associated neurological disease in the era of highly active antiretroviral therapy. J. Neurovirol..

[6] Evans S.R., Ellis R.J., Chen H., Yeh T., Lee A.J., Schifitto G., Wu K., Bosch R.J., McArthur J.C., Simpson D.M. (2011). Peripheral neuropathy in HIV: Prevalence and risk factors. AIDS.

[7] Simpson D.M., Kitch D., Evans S.R., McArthur J.C., Asmuth D.M., Cohen B., Goodkin K., Gerschenson M., So Y., Marra C.M. (2006). HIV neuropathy natural history cohort study: Assessment measures and risk factors. Neurology.

[8] Nakamoto B.K., McMurtray A., Davis J., Valcour V., Watters M.R., Shiramizu B., Chow D.C., Kallianpur K., Shikuma C.M. (2010). Incident neuropathy in HIV-infected patients on HAART. AIDS Res. Hum. Retrovir..

[9] Shah A., Gangwani M.R., Chaudhari N.S., Glazyrin A., Bhat H.K., Kumar A. (2016). Neurotoxicity in the Post-HAART Era: Caution for the Antiretroviral Therapeutics. Neurotox. Res..

[10] Eggleton J.S., Nagalli S. (2021). Highly Active Antiretroviral Therapy (HAART). StatPearls.

[11] Robinson B., Li Z., Nath A. (2007). Nucleoside reverse transcriptase inhibitors and human immunodeficiency virus proteins cause axonal injury in human dorsal root ganglia cultures. J. Neurovirol..

[12] Wu T., Zhang J., Geng M., Tang S.-J., Zhang W., Shu J. (2017). Nucleoside reverse transcriptase inhibitors (NRTIs) induce proinflammatory cytokines in the CNS via Wnt5a signaling. Sci. Rep..

[13] Dorr P., Westby M., Dobbs S., Griffin P., Irvine B., Macartney M., Mori J., Rickett G., Smith-Burchnell C., Napier C. (2005). Maraviroc (UK-427,857), a potent, orally bioavailable, and selective small-molecule inhibitor of chemokine receptor CCR5 with broad-spectrum anti-human immunodeficiency virus type 1 activity. Antimicrob. Agents Chemother..

[14] Oldfield V., Keating G.M., Plosker G. (2005). Enfuvirtide: A review of its use in the management of HIV infection. Drugs.

[15] Cherry C.L., Duncan A.J., Mackie K.F., Wesselingh S.L., Brew B.J. (2008). A report on the effect of commencing enfuvirtide on peripheral neuropathy. AIDS Res. Hum. Retrovir..

[16] Emu B., Fessel J., Schrader S., Kumar P., Richmond G., Win S., Weinheimer S., Marsolais C., Lewis S. (2018). Phase 3 Study of Ibalizumab for Multidrug-Resistant HIV-1. N. Engl. J. Med..

[17] Kaur M., Rawal R.K., Rath G., Goyal A.K. (2018). Structure Based Drug Design: Clinically Relevant HIV-1 Integrase Inhibitors. Curr. Top. Med. Chem..

[18] Teppler H., Brown D.D., Leavitt R.Y., Sklar P., Wan H., Xu X., Lievano F., Lehman H.P., Mast T.C., Nguyen B.-Y.T. (2011). Long-term safety from the raltegravir clinical development program. Curr. HIV Res..

[19] Fettiplace A., Stainsby C., Winston A., Givens N., Puccini S., Vannappagari V., Hsu R., Fusco J., Quercia R., Aboud M. (2017). Psychiatric Symptoms in Patients Receiving Dolutegravir. J. Acquir. Immune Defic. Syndr..

[20] Blas-García A., Polo M., Alegre F., Funes H.A., Martínez E., Apostolova N., Esplugues J.V. (2014). Lack of mitochondrial toxicity of darunavir, raltegravir and rilpivirine in neurons and hepatocytes: A comparison with efavirenz. J. Antimicrob. Chemother..

[21] Latronico T., Pati I., Ciavarella R., Fasano A., Mengoni F., Lichtner M., Vullo V., Mastroianni C.M., Liuzzi G.M. (2018). In vitro effect of antiretroviral drugs on cultured primary astrocytes: Analysis of neurotoxicity and matrix metalloproteinase inhibition. J. Neurochem..

[22] Montenegro-Burke J.R., Woldstad C.J., Fang M., Bade A.N., McMillan J., Edagwa B., Boska M.D., Gendelman H.E., Siuzdak G. (2019). Nanoformulated Antiretroviral Therapy Attenuates Brain Metabolic Oxidative Stress. Mol. Neurobiol..

[23] Lv Z., Chu Y., Wang Y. (2015). HIV protease inhibitors: A review of molecular selectivity and toxicity. HIVAIDS.

[24] Jensen B.K., Monnerie H., Mannell M.V., Gannon P.J., Espinoza C.A., Erickson M.A., Bruce-Keller A.J., Gelman B.B., Briand L.A., Pierce R.C. (2015). Altered Oligodendrocyte Maturation and Myelin Maintenance: The Role of Antiretrovirals in HIV-Associated Neurocognitive Disorders. J. Neuropathol. Exp. Neurol..

[25] Gupta S., Knight A.G., Losso B.Y., Ingram D.K., Keller J.N., Bruce-Keller A.J. (2012). Brain injury caused by HIV protease inhibitors: Role of lipodystrophy and insulin resistance. Antivir. Res..

[26] Pettersen J.A., Jones G., Worthington C., Krentz H.B., Keppler O.T., Hoke A., Gill M.J., Power C. (2006). Sensory neuropathy in human immunodeficiency virus/acquired immunodeficiency syndrome patients: Protease inhibitor-mediated neurotoxicity. Ann. Neurol..

[27] Ellis R.J., Marquie-Beck J., Delaney P., Alexander T., Clifford D.B., McArthur J.C., Simpson D.M., Ake C., Collier A.C., Gelman B.B. (2008). Human immunodeficiency virus protease inhibitors and risk for peripheral neuropathy. Ann. Neurol..

[28] Eastone J.A., Decker C.F. (1997). New-onset diabetes mellitus associated with use of protease inhibitor. Ann. Intern. Med..

[29] Morlese J.F., Qazi N.A., Gazzard B.G., Nelson M.R. (2002). Nevirapine-induced neuropsychiatric complications, a class effect of non-nucleoside reverse transcriptase inhibitors?. AIDS.

[30] Wise M.E.J., Mistry K., Reid S. (2002). Drug points: Neuropsychiatric complications of nevirapine treatment. BMJ.

[31] Arendt G., de Nocker D., von Giesen H.-J., Nolting T. (2007). Neuropsychiatric side effects of efavirenz therapy. Expert Opin. Drug Saf..

[32] Kenedi C.A., Goforth H.W. (2011). A systematic review of the psychiatric side-effects of efavirenz. AIDS Behav..

[33] Apostolova N., Funes H.A., Blas-Garcia A., Galindo M.J., Alvarez A., Esplugues J.V. (2015). Efavirenz and the CNS: What we already know and questions that need to be answered. J. Antimicrob. Chemother..

[34] O’Mahony S.M., Myint A.-M., Steinbusch H., Leonard B.E. (2005). Efavirenz induces depressive-like behaviour, increased stress response and changes in the immune response in rats. Neuroimmunomodulation.

[35] Bienstock R.J., Copeland W.C. (2004). Molecular insights into NRTI inhibition and mitochondrial toxicity revealed from a structural model of the human mitochondrial DNA polymerase. Mitochondrion.

[36] Yuan S., Shi Y., Guo K., Tang S.-J. (2018). Nucleoside Reverse Transcriptase Inhibitors (NRTIs) Induce Pathological Pain through Wnt5a-Mediated Neuroinflammation in Aging Mice. J. Neuroimmune Pharmacol..

[37] Dalakas M.C. (2001). Peripheral neuropathy and antiretroviral drugs. J. Peripher. Nerv. Syst. JPNS.

[38] Dalakas M.C., Semino-Mora C., Leon-Monzon M. (2001). Mitochondrial alterations with mitochondrial DNA depletion in the nerves of AIDS patients with peripheral neuropathy induced by 2′3′-dideoxycytidine (ddC). Lab. Investig. J. Tech. Methods Pathol..

[39] Berger A.R., Arezzo J.C., Schaumburg H.H., Skowron G., Merigan T., Bozzette S., Richman D., Soo W. (1993). 2′,3′-dideoxycytidine (ddC) toxic neuropathy: A study of 52 patients. Neurology.

[40] Drake S.M. (2000). NNRTIs-a new class of drugs for HIV. J. Antimicrob. Chemother..

[41] Srivastava A., Birari V., Sinha S. (2020). Small Conformational Changes Underlie Evolution of Resistance to NNRTI in HIV Reverse Transcriptase. Biophys. J..

[42] Margolis A.M., Heverling H., Pham P.A., Stolbach A. (2014). A review of the toxicity of HIV medications. J. Med. Toxicol. Off. J. Am. Coll. Med. Toxicol..

[43] De Luca A., Pezzotti P., Boucher C., Döring M., Incardona F., Kaiser R., Lengauer T., Pfeifer N., Schülter E., Vandamme A.-M. (2019). Clinical use, efficacy, and durability of maraviroc for antiretroviral therapy in routine care: A European survey. PLoS ONE.

[44] Miao M., De Clercq E., Li G. (2020). Clinical significance of chemokine receptor antagonists. Expert Opin. Drug Metab. Toxicol..

[45] Marr P., Walmsley S. (2008). Reassessment of enfuvirtide’s role in the management of HIV-1 infection. Expert Opin. Pharmacother..

[46] Beccari M.V., Mogle B.T., Sidman E.F., Mastro K.A., Asiago-Reddy E., Kufel W.D. (2019). Ibalizumab, a Novel Monoclonal Antibody for the Management of Multidrug-Resistant HIV-1 Infection. Antimicrob. Agents Chemother..

[47] Blair H.A. (2020). Ibalizumab: A Review in Multidrug-Resistant HIV-1 Infection. Drugs.

[48] Hazuda D.J., Felock P., Witmer M., Wolfe A., Stillmock K., Grobler J.A., Espeseth A., Gabryelski L., Schleif W., Blau C. (2000). Inhibitors of strand transfer that prevent integration and inhibit HIV-1 replication in cells. Science.

[49] Schafer J.J., Squires K.E. (2010). Integrase inhibitors: A novel class of antiretroviral agents. Ann. Pharmacother..

[50] Charpentier C., Descamps D. (2018). Resistance to HIV Integrase Inhibitors: About R263K and E157Q Mutations. Viruses.

[51] Engelman A.N. (2019). Multifaceted HIV integrase functionalities and therapeutic strategies for their inhibition. J. Biol. Chem..

[52] Kumar G.N., Rodrigues A.D., Buko A.M., Denissen J.F. (1996). Cytochrome P450-mediated metabolism of the HIV-1 protease inhibitor ritonavir (ABT-538) in human liver microsomes. J. Pharmacol. Exp. Ther..

[53] Rublein J.C., Eron J.J., Butts J.D., Raasch R.H. (1999). Discontinuation rates for protease inhibitor regimens containing ritonavir 600 mg versus ritonavir 400 mg plus saquinavir 400 mg. Ann. Pharmacother..

[54] D’Arminio Monforte A., Lepri A.C., Rezza G., Pezzotti P., Antinori A., Phillips A.N., Angarano G., Colangeli V., De Luca A., Ippolito G. (2000). Insights into the reasons for discontinuation of the first highly active antiretroviral therapy (HAART) regimen in a cohort of antiretroviral naïve patients. I.CO.N.A. Study Group. Italian Cohort of Antiretroviral-Naïve Patients. AIDS.

[55] Carr A., Cooper D.A. (2000). Adverse effects of antiretroviral therapy. Lancet.

[56] Brown T.T., Cole S.R., Li X., Kingsley L.A., Palella F.J., Riddler S.A., Visscher B.R., Margolick J.B., Dobs A.S. (2005). Antiretroviral therapy and the prevalence and incidence of diabetes mellitus in the multicenter AIDS cohort study. Arch. Intern. Med..

[57] Grilo N.M., João Correia M., Miranda J.P., Cipriano M., Serpa J., Matilde Marques M., Monteiro E.C., Antunes A.M.M., Diogo L.N., Pereira S.A. (2017). Unmasking efavirenz neurotoxicity: Time matters to the underlying mechanisms. Eur. J. Pharm. Sci. Off. J. Eur. Fed. Pharm. Sci..

[58] Eiseman J.L., Yetter R.A., Fredrickson T.N., Shapiro S.G., MacAuley C., Bilello J.A. (1991). Effect of 3′azidothymidine administered in drinking water or by continuous infusion on the development of MAIDS. Antiviral Res..

[59] De la Asunción J.G., del Olmo M.L., Sastre J., Pallardó F.V., Viña J. (1999). Zidovudine (AZT) causes an oxidation of mitochondrial DNA in mouse liver. Hepatology.

[60] Jirkof P., Durst M., Klopfleisch R., Palme R., Thöne-Reineke C., Buttgereit F., Schmidt-Bleek K., Lang A. (2019). Administration of Tramadol or Buprenorphine via the drinking water for post-operative analgesia in a mouse-osteotomy model. Sci. Rep..

[61] Polydefkis M., Yiannoutsos C.T., Cohen B.A., Hollander H., Schifitto G., Clifford D.B., Simpson D.M., Katzenstein D., Shriver S., Hauer P. (2002). Reduced intraepidermal nerve fiber density in HIV-associated sensory neuropathy. Neurology.

[62] Munawar N., Oriowo M.A., Masocha W. (2017). Antihyperalgesic Activities of Endocannabinoids in a Mouse Model of Antiretroviral-Induced Neuropathic Pain. Front. Pharmacol..

[63] Aly E., Khajah M.A., Masocha W. (2019). β-Caryophyllene, a CB2-Receptor-Selective Phytocannabinoid, Suppresses Mechanical Allodynia in a Mouse Model of Antiretroviral-Induced Neuropathic Pain. Molecules.

[64] Sanna M.D., Manassero G., Vercelli A., Herdegen T., Galeotti N. (2020). The isoform-specific functions of the c-Jun N-terminal kinase (JNK) in a mouse model of antiretroviral-induced painful peripheral neuropathy. Eur. J. Pharmacol..

[65] Dubinsky R.M., Yarchoan R., Dalakas M., Broder S. (1989). Reversible axonal neuropathy from the treatment of AIDS and related disorders with 2′,3′-dideoxycytidine (ddC). Muscle Nerve.

[66] Deuis J.R., Dvorakova L.S., Vetter I. (2017). Methods Used to Evaluate Pain Behaviors in Rodents. Front. Mol. Neurosci..

[67] Tieu L., Boomhower B., George O. (2020). Hot Water Tail Immersion Test. https://protocols.io/view/hot-water-tail-immersion-test-bhxbj7in.

[68] Polydefkis M. (2006). Skin biopsy findings predict development of symptomatic neuropathy in patients with HIV. Nat. Clin. Pract. Neurol..

[69] Obermann M., Katsarava Z., Esser S., Sommer C., He L., Selter L., Yoon M.-S., Kaube H., Diener H.-C., Maschke M. (2008). Correlation of epidermal nerve fiber density with pain-related evoked potentials in HIV neuropathy. Pain.

[70] Patel I.G., Kamerman P.R. (2019). Colocalization of pain and reduced intraepidermal nerve fiber density in individuals with HIV-associated sensory neuropathy. Pain Rep..

[71] Lanman T., Letendre S., Ma Q., Bang A., Ellis R. (2021). CNS Neurotoxicity of Antiretrovirals. J. Neuroimmune Pharmacol..

[72] Reyns T., De Boever S., Schauvliege S., Gasthuys F., Meissonnier G., Oswald I., De Backer P., Croubels S. (2009). Influence of administration route on the biotransformation of amoxicillin in the pig. J. Vet. Pharmacol. Ther..

[73] Fernandez E., Perez R., Hernandez A., Tejada P., Arteta M., Ramos J.T. (2011). Factors and Mechanisms for Pharmacokinetic Differences between Pediatric Population and Adults. Pharmaceutics.

[74] Atkinson H.C., Stanescu I., Frampton C., Salem I.I., Beasley C.P.H., Robson R. (2015). Pharmacokinetics and Bioavailability of a Fixed-Dose Combination of Ibuprofen and Paracetamol after Intravenous and Oral Administration. Clin. Drug Investig..

[75] von Richter O., Lemke L., Haliduola H., Fuhr R., Koernicke T., Schuck E., Velinova M., Skerjanec A., Poetzl J., Jauch-Lembach J. (2019). GP2017, an adalimumab biosimilar: Pharmacokinetic similarity to its reference medicine and pharmacokinetics comparison of different administration methods. Expert Opin. Biol. Ther..

[76] Al Shoyaib A., Archie S.R., Karamyan V.T. (2019). Intraperitoneal Route of Drug Administration: Should it Be Used in Experimental Animal Studies?. Pharm. Res..

[77] Jeong S.-H., Jang J.-H., Lee Y.-B. (2020). Pharmacokinetic Comparison of Three Different Administration Routes for Topotecan Hydrochloride in Rats. Pharmaceuticals.

[78] Alagga A.A., Gupta V. (2021). Drug Absorption. StatPearls.

[79] Rathbun R.C., Rossi D.R. (2002). Low-dose ritonavir for protease inhibitor pharmacokinetic enhancement. Ann. Pharmacother..

[80] Huang W., Calvo M., Pheby T., Bennett D.L., Rice A.S. (2017). A rodent model of HIV protease inhibitor indinavir induced peripheral neuropathy. Pain.

[81] Stern A.L., Lee R.N., Panvelker N., Li J., Harowitz J., Jordan-Sciutto K.L., Akay-Espinoza C. (2018). Differential Effects of Antiretroviral Drugs on Neurons In Vitro: Roles for Oxidative Stress and Integrated Stress Response. J. Neuroimmune Pharmacol..

[82] Letendre S.L., Mills A.M., Tashima K.T., Thomas D.A., Min S.S., Chen S., Song I.H., Piscitelli S.C., extended ING116070 study team (2014). ING116070: A study of the pharmacokinetics and antiviral activity of dolutegravir in cerebrospinal fluid in HIV-1-infected, antiretroviral therapy-naive subjects. Clin. Infect. Dis. Off. Publ. Infect. Dis. Soc. Am..

[83] Piotrowska A., Kwiatkowski K., Rojewska E., Makuch W., Mika J. (2016). Maraviroc reduces neuropathic pain through polarization of microglia and astroglia—Evidence from in vivo and in vitro studies. Neuropharmacology.

[84] Lyseng-Williamson K.A., Reynolds N.A., Plosker G.L. (2005). Tenofovir disoproxil fumarate: A review of its use in the management of HIV infection. Drugs.

[85] Pillay P., Wadley A.L., Cherry C.L., Karstaedt A.S., Kamerman P.R. (2019). Clinical diagnosis of sensory neuropathy in HIV patients treated with tenofovir: A 6-month follow-up study. J. Peripher. Nerv. Syst. JPNS.

[86] Chang H., Wang Y., Wu H., Nathans J. (2014). Flat Mount Imaging of Mouse Skin and Its Application to the Analysis of Hair Follicle Patterning and Sensory Axon Morphology. J. Vis. Exp. JoVE.

[87] Nguyen M.B., Valdes V.J., Cohen I., Pothula V., Zhao D., Zheng D., Ezhkova E. (2019). Dissection of Merkel cell formation in hairy and glabrous skin reveals a common requirement for FGFR2-mediated signalling. Exp. Dermatol..

[88] Herzberg U., Sagen J. (2001). Peripheral nerve exposure to HIV viral envelope protein gp120 induces neuropathic pain and spinal gliosis. J. Neuroimmunol..

[89] Yuan S.B., Shi Y., Chen J., Zhou X., Li G., Gelman B.B., Lisinicchia J.G., Carlton S.M., Ferguson M.R., Tan A. (2014). Gp120 in the pathogenesis of human immunodeficiency virus-associated pain. Ann. Neurol..

[90] Wodarski R., Bagdas D., Paris J.J., Pheby T., Toma W., Xu R., Damaj M.I., Knapp P.E., Rice A.S.C., Hauser K.F. (2018). Reduced intraepidermal nerve fibre density, glial activation, and sensory changes in HIV type-1 Tat-expressing female mice: Involvement of Tat during early stages of HIV-associated painful sensory neuropathy. Pain Rep..

[91] Bagdas D., Paris J.J., Carper M., Wodarski R., Rice A.S.C., Knapp P.E., Hauser K.F., Damaj M.I. (2020). Conditional expression of HIV-1 tat in the mouse alters the onset and progression of tonic, inflammatory and neuropathic hypersensitivity in a sex-dependent manner. Eur. J. Pain.

[92] Shi Y., Yuan S., Tang S.-J. (2019). Morphine and HIV-1 gp120 cooperatively promote pathogenesis in the spinal pain neural circuit. Mol. Pain.

[93] Cherry C.L., Affandi J.S., Imran D., Yunihastuti E., Smyth K., Vanar S., Kamarulzaman A., Price P. (2009). Age and height predict neuropathy risk in patients with HIV prescribed stavudine. Neurology.

[94] Kaku M., Simpson D.M. (2014). HIV neuropathy. Curr. Opin. HIV AIDS.

[95] Thakur K.T., Boubour A., Saylor D., Das M., Bearden D.R., Birbeck G.L. (2019). Global HIV neurology: A comprehensive review. AIDS.

